# Supplementary material for: Can routine assessment of older people’s mental health lead to improved outcomes: A regression discontinuity analysis
Source: PLoS One. 2024 Mar 19;19(3):e0300651. doi: 10.1371/journal.pone.0300651 (PMC10950230; doi:10.1371/journal.pone.0300651)
Supplement: S1 Fig — (DOCX) [file pone.0300651.s001.docx]

**Geriatric Depression Scale (Short Form) Self-Rated Version**

Patient’s Name: Date:

***Instructions: Choose the best answer for how you felt over the past week.***

| **No.** | **Question** | **Answer** | **Score** |
| --- | --- | --- | --- |
| 1. | Are you basically satisfied with your life? | YES / NO |  |
| 2. | Have you dropped many of your activities and interests? | YES / NO |  |
| 3. | Do you feel that your life is empty? | YES / NO |  |
| 4. | Do you often get bored? | YES / NO |  |
| 5. | Are you in good spirits most of the time? | YES / NO |  |
| 6. | Are you afraid that something bad is going to happen to you? | YES / NO |  |
| 7. | Do you feel happy most of the time? | YES / NO |  |
| 8. | Do you often feel helpless? | YES / NO |  |
| 9. | Do you prefer to stay at home, rather than going out and doing new things? | YES / NO |  |
| 10. | Do you feel you have more problems with memory than most people? | YES / NO |  |
| 11. | Do you think it is wonderful to be alive? | YES / NO |  |
| 12. | Do you feel pretty worthless the way you are now? | YES / NO |  |
| 13. | Do you feel full of energy? | YES / NO |  |
| 14. | Do you feel that your situation is hopeless? | YES / NO |  |
| 15. | Do you think that most people are better off than you are? | YES / NO |  |
| TOTAL | | |  |

(Sheikh & Yesavage, 1986)
