# Supplementary material for: Can routine assessment of older people’s mental health lead to improved outcomes: A regression discontinuity analysis
Source: PLoS One. 2024 Mar 19;19(3):e0300651. doi: 10.1371/journal.pone.0300651 (PMC10950230; doi:10.1371/journal.pone.0300651)
Supplement: Attachment — Submitted filename: renamed_7798e.docx [file pone.0300651.s002.docx]

# COMMENTS – Manuscript PONE-D-23-04252

Title: “Can routine assessment of older people’s mental health lead to improved outcomes: A regression discontinuity analysis”

**Important note: This review pertains only to ‘statistical aspects’ of the study and so ‘clinical aspects’ [like medical importance, relevance of the study, ‘clinical significance and implication(s)’ of the whole study, etc.] are to be evaluated [should be assessed] separately/independently. Further please note that any ‘statistical review’ is generally done under the assumption that (such) study specific methodological [as well as execution] issues are perfectly taken care of by the investigator(s). This review is not an exception to that and so does not cover clinical aspects {however, seldom comments are made only if those issues are intimately / scientifically related & intermingle with ‘statistical aspects’ of the study}. Agreed that ‘statistical methods’ are used as just tools here, however, they are vital part of methodology [and so should be given due importance]. I look at the manuscript in/with statistical view point, other reviewer(s) look(s) at it with different angle so that in totality the review is very comprehensive. However, there should be efforts from authors side to improve (may be by taking clues from reviewer’s comments). Therefore, please do not limit the revision only (with respect) to comments made here.**

**COMMENTS:** Although this manuscript is well drafted [and the study is excellent with respect to most of the aspects], I have few observations/concerns (different opinion) which are given below:

Firstly, {although on one hand I must congratulate and appreciate authors having used this design} it may please be noted that the RD (regression discontinuity) design lacks the random assignment feature. In addition to this, there are several issues* that disallow a status equal to the randomized experiment and the RD design has to be classified as a quasi-experimental design.

Few of the issues described in literature are:

- it is a new design and not yet as clear as the randomized experiment.
- the RD design heavily depends on correctly modeling the functional form of the relationship between the assignment variable and the outcome variable. Therefore, in order to obtain unbiased estimates, more sophisticated statistical analyses are required than is usual in experimental settings.
- the RD design has less power than the randomized experiment. This is mainly due to [multicollinearity](https://www.sciencedirect.com/topics/mathematics/multicollinearity) between the assignment and treatment variable. The loss of power is greater the more the cutoff deviates from the mean of the assignment variable. When nonlinear effects (e.g., interaction and quadratic effects) are present, multicollinearity increases as well as reduces the power of the design. In order to increase the power to 0.80, the RD design needs up to 3 times as many participants as a randomized experiment

Though sometimes the argument(s) made that “from a methodological point of view, inferences which are drawn from a well-implemented RD design are comparable in internal validity to conclusions from randomized experiments” it is not very convincing. It is often said that, in principle the RD design is as strong in internal validity as its randomized experimental alternatives, however, in practice, the validity of the RD design depends directly on how well the analyst can model the true pre-post relationship, certainly a nontrivial statistical problem.

The real allure of the RD Design is that it allows us to assign the treatment or program to those who most need or deserve it. Thus, the real attractiveness of the design is ethical – we don’t have to deny the program or treatment to participants who might need it as we do in randomized studies.

With this background information I have, few questions are raised in mind [though they may appear to be silly on prima-facie, you need to clarify these things because mind you that this is a scientific/academic document and so all details should be clearly/correctly communicated (do not take readers’ for granted)].

- As per line 26 [Design] data are from a randomised controlled trial. My question is {though in lines 142-143 it is stated that adjustment for covariates included that for participant’s REFORM trial allocation}, participant’s those who are randomly allocated to intervention arm of REFORM trial are already influenced/affected by that intervention [those who are randomly allocated to control arm (even if active control or placebo) of REFORM trial are already influenced/affected differently] then will it likely to affect your outcome.
- There may be substantial period between ‘baseline’ reading time and your intervention. What about that?

Section ‘**Study Design and Statistical Methods**’ in lines 106 onwards is fairly well drafted but authors may think including above (and/or other such) points. I agree with the ‘Limitations’ part highlighted/enumerated in lines 260 onwards [but not so much with ‘Strengths’ part of the section].

Except these minor points, the article is acceptable. However, mind you that as pointed out in ‘important note’ above “This review pertains only to ‘statistical aspects’ of the study and so ‘clinical aspects’ should be assessed separately/independently.
