# Supplementary material for: Can routine assessment of older people’s mental health lead to improved outcomes: A regression discontinuity analysis
Source: PLoS One. 2024 Mar 19;19(3):e0300651. doi: 10.1371/journal.pone.0300651 (PMC10950230; doi:10.1371/journal.pone.0300651)
Supplement: Attachment — Submitted filename: PONE-D-23-04252_reviewer.docx [file pone.0300651.s003.docx]

**PLOS ONE**

**Can routine assessment of older people’s mental health lead to improved outcomes: A regression discontinuity analysis**

--Manuscript Draft--

| **Manuscript Number:** | PONE-D-23-04252 |
| --- | --- |
| **Article Type:** | Research Article |
| **Full Title:** | Can routine assessment of older people’s mental health lead to improved outcomes: A regression discontinuity analysis |
| **Short Title:** | Routine case finding and older people’s mental health |
| **Corresponding Author:** | Kalpita Baird  University of York Department of Health Sciences York, UNITED KINGDOM |
| **Keywords:** | Regression discontinuity; Depression; Case finding; Elderly; General Practice; Geriatrics; Mental Health |
| **Abstract:** | Abstract  Objective: To assess whether case finding for depression among people aged 65 and above improves mental health.  Design: Opportunistic evaluation using a regression discontinuity analysis with data from a randomised controlled trial.  Setting: The REFORM trial, a falls prevention study that recruited patients from NHS podiatry clinics.  Participants: 1010 community-dwelling adults over the age of 65 with at least one risk factor for falling (recent previous fall or fear of falling).  Intervention: Letter sent to patient’s General Practitioner if they scored 10 points or above on the 15-item Geriatric Depression Scale (GDS-15) informing them of the patient’s risk of depression.  Main outcome measure: GDS-15 score six months after initial completion of GDS-15. Results: 895 (88.6%) of the 1010 participants randomised into REFORM had a valid baseline and six-month GDS-15 score and were included in this study. The mean GDS-15 baseline score was 3.5 (SD 3.0, median 3.0, range 0-15); 639 (71.4%) scored  0-4, 204 (22.8%) scored 5-9 indicating mild depression, and 52 (5.8%) scored 10 or higher indicating severe depression. At six months follow-up, those scoring 10 points or higher at baseline had, on average, a reduction of 1.08 points on the GDS-15 scale (95% confidence interval -1.83 to -0.33, p=0.005) compared to those scoring less than 10, using the simplest linear regression model.  Conclusion: Case finding of depression in podiatry patients based on a GDS-15 score of 10 or more followed by a letter to their General Practitioner significantly reduced depression severity. Whether this applies to all older patients in primary care is unknown. Further research is required to confirm these findings. Regression discontinuity analyses could be prespecified and embedded within other existing research studies. |
| **Order of Authors:** | Kalpita Baird |
|  | Ailish Byrne |
|  | Sarah Cockayne |
|  | Rachel Cunningham-Burley |
|  | Caroline Fairhurst |
|  | Joy Adamson |
|  | Wesley Vernon |
|  | David Torgerson |
| **Additional Information:** |  |
| **Question** | **Response** |

| **Financial Disclosure**  Enter a financial disclosure statement that describes the sources of funding for the work included in this submission. Review the [submission guidelines](http://journals.plos.org/plosone/s/submission-guidelines#loc-financial-disclosure-statement) for detailed requirements. View published research articles from [*PLOS ONE*](http://journals.plos.org/plosone/) for specific examples.  This statement is required for submission and **will appear in the published article** if the submission is accepted. Please make sure it is accurate. | The author(s)s received no specific funding for this work. However, the original REFORM trial was funded by the National Institute for Health Research (NIHR) Health Technology Assessment (HTA) Programme (Programme grant number 09/77/01). |
| --- | --- |
| **Unfunded studies**  Enter: *The author(s) received no specific funding for this work.*  **Funded studies**  Enter a statement with the following details:   - Initials of the authors who received each award - Grant numbers awarded to each author - The full name of each funder - URL of each funder website - Did the sponsors or funders play any role in the study design, data collection and analysis, decision to publish, or preparation of the manuscript? - **NO** - Include this sentence at the end of your statement: *The funders had no role in study design, data collection and analysis, decision to publish, or preparation of the manuscript.* - **YES** - Specify the role(s) played. |  |
| * typeset |  |
| **Competing Interests**  Use the instructions below to enter a competing interest statement for this submission. On behalf of all authors, disclose any [competing interests](http://journals.plos.org/plosone/s/competing-interests) that could be perceived to bias this work—acknowledging all financial support and any other relevant financial or non- financial competing interests.  This statement is required for submission | I have read the journal's policy and the authors of this manuscript have the following competing interests: SC, CF, JA, WV, and DT received funding from the National Institute for Health Research (NIHR) Health Technology Assessment (HTA) paid to their employer, University of York, for the REFORM trial. All other authors declare no competing interests. |

| and **will appear in the published article** if the submission is accepted. Please make sure it is accurate and that any funding sources listed in your Funding Information later in the submission form are also declared in your Financial Disclosure statement.  View published research articles from  [*PLOS ONE*](http://journals.plos.org/plosone/) for specific examples. |  |
| --- | --- |
| **NO authors have competing interests**  Enter: *The authors have declared that no competing interests exist.*  **Authors with competing interests**  Enter competing interest details beginning with this statement:  *I have read the journal's policy and the authors of this manuscript have the following competing interests: [insert competing interests here]* |  |
| * typeset |  |
| **Ethics Statement**  Enter an ethics statement for this submission. This statement is required if the study involved:   - Human participants - Human specimens or tissue - Vertebrate animals or cephalopods - Vertebrate embryos or tissues - Field research   Write "N/A" if the submission does not require an ethics statement.  General guidance is provided below. Consult the [submission guidelines](http://journals.plos.org/plosone/s/submission-guidelines#loc-guidelines-for-specific-study-types) for detailed instructions. **Make sure that all information entered here is included in the Methods section of the manuscript.** | This study used deidentified data. The REFORM study was approved by the East of England National Research Ethics Committee (Cambridge East) on the 09.11.2011 and all participants provided written informed consent. |

| **Format for specific study types**  **Human Subject Research (involving human participants and/or tissue)**   - Give the name of the institutional review board or ethics committee that approved the study - Include the approval number and/or a statement indicating approval of this research - Indicate the form of consent obtained (written/oral) or the reason that consent was not obtained (e.g. the data were analyzed anonymously)   **Animal Research (involving vertebrate animals, embryos or tissues)**   - Provide the name of the Institutional Animal Care and Use Committee (IACUC) or other relevant ethics board that reviewed the study protocol, and indicate whether they approved this research or granted a formal waiver of ethical approval - Include an approval number if one was obtained - If the study involved *non-human primates*, add *additional details* about animal welfare and steps taken to ameliorate suffering - If anesthesia, euthanasia, or any kind of animal sacrifice is part of the study, include briefly which substances and/or methods were applied   **Field Research**  Include the following details if this study involves the collection of plant, animal, or other materials from a natural setting:   - Field permit number - Name of the institution or relevant body that granted permission |  |
| --- | --- |
|  |  |
| **Data Availability**  Authors are required to make all data underlying the findings described fully available, without restriction, and from the time of publication. PLOS allows rare exceptions to address legal and ethical concerns. See the [PLOS Data Policy](http://journals.plos.org/plosone/s/data-availability) and [FAQ](http://journals.plos.org/plosone/s/data-availability#loc-faqs-for-data-policy) for detailed information. | No - some restrictions will apply |

| A Data Availability Statement describing where the data can be found is required at submission. Your answers to this question constitute the Data Availability Statement and **will be published in the article**, if accepted. |  |
| --- | --- |
| **Important:** Stating ‘data available on request from the author’ is not sufficient. If your data are only available upon request, select ‘No’ for the first question and explain your exceptional situation in the text box. |  |
| Do the authors confirm that all data underlying the findings described in their manuscript are fully available without restriction? |  |
| **Describe where the data may be found in full sentences. If you are copying our sample text, replace any instances of XXX with the appropriate details.**   - If the data are **held or will be held in a public repository**, include URLs, accession numbers or DOIs. If this information will only be available after acceptance, indicate this by ticking the box below. For example: *All XXX files are available from the XXX database (accession number(s) XXX, XXX.*). - If the data are all contained **within the manuscript and/or Supporting Information files**, enter the following: *All relevant data are within the manuscript and its Supporting Information files.* - If neither of these applies but you are able to provide **details of access elsewhere**, with or without limitations, please do so. For example:   *Data cannot be shared publicly because of [XXX]. Data are available from the XXX Institutional Data Access / Ethics Committee (contact via XXX) for researchers who meet the criteria for access to confidential data.*  *The data underlying the results presented in the study are available from (include the name of the third party* | Data are available upon reasonable request. All data requests, with statistical analysis plans, should be made to the corresponding author and will be considered on a case- by-case basis by the authors. All data requests will be managed in accordance with York Trials Unit, University of York, standard operating procedures. |

| *and contact information or URL).*   - This text is appropriate if the data are owned by a third party and authors do not have permission to share the data.   * typeset |  |
| --- | --- |
| Additional data availability information: |  |

Manuscript	[Click here to access/download;Manuscript;GDS PAPER 20230213.docx](https://www.editorialmanager.com/pone/download.aspx?id=32654588&guid=87e6f512-3a2a-4eea-85b3-eb09f4caf3a0&scheme=1)

# 1 Full Title: Can routine assessment of older people’s mental health lead to improved outcomes: A

2 **regression discontinuity analysis**

# 3 Short Title: Routine case finding and older people’s mental health

4

# 5 Author Details

6 (Kalpita Baird^1^, Ailish Byrne^1^, Sarah Cockayne^1^, Rachel Cunningham-Burley^1^, Caroline Fairhurst^1^, Joy
7 Adamson^1^, Wesley Vernon^2^, David J Torgerson^1^ on behalf of the REFORM trial)
8 Kalpita Baird, MSc, Statistician
9 Ailish Byrne, MPsych, Trial Support Officer
10 Sarah Cockayne, MSc, Research Fellow
11 Rachel Cunningham-Burley, MPH, Research Fellow
12 Caroline Fairhurst, MSc, Statistician
13 Joy Adamson, PhD, Chair in Surgical Trials and Health Sciences
14 Wesley Vernon, PhD, Visiting Professor, University of Huddersfield
15 Professor David Torgerson, PhD, Co-Director
16 ^1^York Trials Unit, Department of Health Sciences, University of York, York, UK
17 ^2^University of Huddersfield, Huddersfield, UK
18 Corresponding Author*: Kalpita Baird; email: [kalpita.baird@york.ac.uk](mailto:kalpita.baird@york.ac.uk)

19

20

21

22

# 23 Abstract

24 **Objective:** To assess whether case finding for depression among people aged 65 and above improves
25 mental health.
26 **Design:** Opportunistic evaluation using a regression discontinuity analysis with data from a
27 randomised controlled trial.
28 **Setting:** The REFORM trial, a falls prevention study that recruited patients from NHS podiatry clinics.
29 **Participants:** 1010 community-dwelling adults over the age of 65 with at least one risk factor for
30 falling (recent previous fall or fear of falling).
31 **Intervention:** Letter sent to patient’s General Practitioner if they scored 10 points or above on the
32 15-item Geriatric Depression Scale (GDS-15) informing them of the patient’s risk of depression.
33 **Main outcome measure:** GDS-15 score six months after initial completion of GDS-15.
34 **Results:** 895 (88.6%) of the 1010 participants randomised into REFORM had a valid baseline and six-
35 month GDS-15 score and were included in this study. The mean GDS-15 baseline score was 3.5 (SD

36	3.0, median 3.0, range 0-15); 639 (71.4%) scored 0-4, 204 (22.8%) scored 5-9 indicating mild

37 depression, and 52 (5.8%) scored 10 or higher indicating severe depression. At six months follow-up,
38 those scoring 10 points or higher at baseline had, on average, a reduction of 1.08 points on the GDS-
39 15 scale (95% confidence interval -1.83 to -0.33, p=0.005) compared to those scoring less than 10,
40 using the simplest linear regression model.
41 **Conclusion:** Case finding of depression in podiatry patients based on a GDS-15 score of 10 or more
42 followed by a letter to their General Practitioner significantly reduced depression severity. Whether
43 this applies to all older patients in primary care is unknown. Further research is required to confirm
44 these findings. Regression discontinuity analyses could be prespecified and embedded within other
45 existing research studies.

# 46 Introduction

47 Depression is the most prevalent mental health disorder amongst the older population [1]. It is
48 estimated that one in four adults over the age of 65 years suffer from depression [2] but
49 approximately 85% of those suffering do not receive help through the National Health Service (NHS)
50 to manage their condition [3]. This may partially reflect patients’ reluctance to disclose depressive
51 symptoms to healthcare providers – potentially linked to a fear of the stigma surrounding mental
52 health disorders [3]. An additional concern is evidence that General Practitioners (GPs) and those in
53 first line contact with this population are faced with difficulties in identifying depressed patients [4,
54 5], in part due to the symptoms of depression amongst older adults varying considerably from those
55 displayed by younger adults [6]. This lack of recognition likely contributes to a significant number of
56 depressed patients failing to receive the help they need from healthcare services.
57 Whilst the United States Preventative Task Force recommends screening for depression in the
58 general adult population [7], routine screening is not currently recommended in the United Kingdom
59 (UK). Rather practitioners are advised to ‘be alert’ to possible depression (particularly in people with
60 a history of depression or a chronic physical health problem) [8]. Furthermore, a large cluster
61 randomised trial evaluating the screening for depression in primary care patients consulting with
62 osteoarthritis found no evidence of a benefit [9].
63 Currently there is no strong evidence for routine case finding of depression among the older
64 population [10]. In this paper we report a natural experiment using a regression discontinuity
65 analysis to assess whether case finding of depression in older adults reduces depressive symptoms
66 six months later.

67

68

69

# 70 Methods

71 This study was an unexpected evaluation resulting from the REFORM randomised controlled trial
72 (RCT) for the prevention of falls. REFORM was a multicentre, two-armed RCT that recruited 1010
73 participants aged 65 and over with a risk factor for falling (recent previous fall or fear of falling) to
74 assess the effectiveness of a podiatry intervention for the prevention of falls in older people [11].
75 Patients were recruited from podiatry clinic lists. The primary outcome was the incidence rate of
76 falls over 12 months; however, secondary outcomes included the proportion of participants
77 presenting with depression at six and 12 months post-randomisation. Within the REFORM trial,
78 depression was classified through the use of the 15-item Geriatric Depression Scale (GDS-15) [12]
79 measured at baseline, six and 12 months. The GDS-15 (see Appendix A) is a popular tool specifically
80 targeted towards detecting depression amongst the older population and is a shortened version of
81 the original 30-item scale [13, 14]. The scale includes items assessing how the responder has felt
82 over the last week, including ‘Are you basically satisfied with your life?’ and ‘Do you feel full of
83 energy?’. A total score of 0-4 is considered normal, 5-9 indicates mild depression, and 10-15
84 indicates severe depression. The validity of the GDS-15 to identify depression within older cohorts,
85 both in its original 30-item and shortened versions, within the UK and cross-culturally, has been
86 displayed consistently across the literature [15-19]. The GDS-15 displays superior or equal levels of
87 specificity, sensitivity, and appropriateness as several other popular depression scales, including the
88 Beck Depression Inventory, Centre for Epidemiologic Studies Depression Scale, Zung Self-rating
89 Depression Scale, and the Hamilton Rating Scale for Depression [20-24].
90 Participants in the REFORM trial completed and returned postal questionnaires to the York Trials
91 Unit to collect participant-reported outcomes, including the GDS-15, at baseline, six and 12 months
92 post-randomisation. On return of the questionnaire, a participant’s GDS-15 score was calculated by
93 hand by a trial coordinator. If a participant scored 10 or more, their consent form was reviewed to
94 confirm whether they had given permission for the study team to contact their GP if any concerns
95 were raised about their health during the course of the study. For those who agreed, an ethically
96 approved letter was sent by second-class post to the participant’s GP, detailing the participant’s
97 GDS-15 score, the date the scale was completed, and information about how to interpret the score.
98 Based on this information, the letter recommended that a consultation with the participant be
99 arranged as soon as practically possible to discuss possible treatment options for their depression
100 that may be available, should the participant choose to take up any help. The trial manager’s contact
101 details and web link to the study protocol were provided in case the GP had any queries. If the GP
102 had been previously notified about a high GDS-15 score for a participant at an earlier trial
103 assessment time point, they were not contacted again, even if the GDS-15 score was 10 or above on
104 a subsequent assessment.
105 105
106 **Study Design and Statistical Methods** – *Regression Discontinuity Design*
107 A regression discontinuity design (RDD) is where a score or threshold is given to a continuous
108 assignment variable and used to determine whether an individual is offered an intervention or not
109 [25, 26]. The continuous assignment variable used for this RDD was the participant’s score at
110 baseline on the GDS-15, where the threshold to receive the intervention (GP contacted) was a score
111 of 10 (or more). The RDD considers the change in score six months later. No other interventions
112 were applied on the basis of the participant’s baseline GDS-15 score.
113 Two versions of RDD exist: ‘sharp’ and ‘fuzzy’. Sharp RDD is deterministic in nature, meaning that all
114 who meet the required threshold or score on the continuous assignment variable are offered the
115 intervention (and those who do not meet the threshold are not); whereas fuzzy RDD is probabilistic,
116 meaning that those who meet the required threshold or score are more likely to be offered the
117 intervention than those who do not [27]. This study used a sharp RDD as 51 (98.1%) of the 52
118 participants who met the required threshold received the intervention. For the remaining
119 participant, a letter was not sent to their GP as they did not consent to this. Given this only applies
120 to one participant, this participant is included in all statistical analyses assuming that they did receive
121 the intervention.
122 A limitation of the RDD is a concern around manipulation of the continuous assignment variable e.g.
123 completion of the measure in such a way as to ensure the cut-point is met or just missed, depending
124 on the context [28]. This is of limited concern here as the GDS-15 is a patient-reported outcome
125 measure and participants were not aware, prior to its completion, that their scoring over a particular
126 threshold on the GDS-15 would trigger a notification letter to their GP with recommendations for a
127 follow-up visit. Manipulation of the assignment variable would therefore be unlikely. However, this
128 was assessed visually using a histogram to check for clustering around the cut-point, which might
129 indicate deliberate attempts to reach, or narrowly avoid, the threshold.
130 In order for the RDD to be valid, certain assumptions must be met [25, 27]. One assumption is that
131 there is continuity in other variables between those who scored slightly above and those who scored
132 slightly below the threshold [27]. This assumption would indicate that participants around the
133 threshold are mainly similar in most other aspects except for the score on the assignment variable
134 and, if met, would allow the estimation of causal effects of the intervention. Continuity amongst
135 other variables around the cut-point was assessed using the methods described by Sood et al. 2014
136 [29]. The observed characteristics of participants that scored eight or nine and those that scored 10
137 or 11 (i.e. those falling just either side of the threshold) were compared, using a t-test or chi-squared
138 test, as appropriate, at the 5% significance level.
139 To conduct the RDD, we considered a parametric approach [28], which uses every observation in the
140 sample. Different functional forms of the regression model were estimated, increasing in complexity:
141 linear, quadratic, cubic, all with and without interaction terms of the GDS-15 at baseline with
142 treatment, and with and without adjusting for further covariates (participant’s age, gender, REFORM
143 trial allocation, and number of prescribed medications at baseline) to improve precision.
144 The simplest form, the linear regression model without an interaction, is:

| 145 | 𝑦_𝑖_ = 𝛼_0_ + 𝛼_1_𝐴𝑏𝑜𝑣𝑒_𝑖_ + 𝛼_2_𝐵𝑎𝑠𝑒𝑙𝑖𝑛𝑒𝐺𝐷𝑆_𝑖_ + 𝜀_𝑖_ | | (1) |
| --- | --- | --- | --- |
| 146 | Where: |  | |
| 147 | • | 𝑦_𝑖_ is participant 𝑖’s score on the GDS-15 at six months | |
| 148 | • | 𝛼_0_ is a constant | |
| 149 | • | 𝜀_𝑖_ is a random error term | |
| 150 | • | 𝐴𝑏𝑜𝑣𝑒_𝑖_ is an indicator variable that is equal to one if participant 𝑖’s score on the GDS-15 at | |
| 151 |  | baseline is 10 or more, and zero otherwise | |
| 152 | • | 𝐵𝑎𝑠𝑒𝑙𝑖𝑛𝑒𝐺𝐷𝑆_𝑖_ is participant 𝑖’s score on the GDS-15 at baseline centred at the cut-point of | |
| 153 |  | 10. | |
| 154 | • | 𝛼_1_, 𝛼_2_ are the regression coefficients. | |

155 In this case, 𝛼_1_ is our estimate of the impact of using the GDS-15 to detect severe depression at
156 baseline on depression severity six-months later.
157 Final model selection was based on minimising the Akaike’s Information Criterion (AIC) and on the
158 results of an F-test. Whilst AIC can demonstrate whether one model fits the data better than
159 another, it does not give an indication of how well the model fits the data overall. For this reason,
160 the F-test approach as described by Lee and Lemieux [28] was also conducted.
161 161

# 162 Public Patient Involvement

163 There was no public or patient involvement for this analysis; however, the REFORM trial as a whole
164 had a patient reference group, comprising of four people who were representative of trial
165 participants, who gave comments on patient-facing material including the questionnaires containing
166 the GDS-15 instrument.
167 167

## 168 Results

169 In total, 895 (88.6%) of the 1010 participants randomised into REFORM had a valid baseline and six-
170 month GDS-15 score and were included in this RDD analysis. Among these 895 participants, the
171 mean GDS-15 baseline score was 3.5 (SD 3.0, median 3.0, range 0-15); 639 (71.4%) scored between 0
172 and 4, 204 (22.8%) between 5 and 9, and 52 (5.8%) between 10 and 15, indicating severe
173 depression. These 52 participants were recruited from 43 GP surgeries (average 1.2 per surgery,
174 range 1 to 3).
175 At six months, the overall mean GDS-15 score was 3.7 (SD 3.1, median 3.0, range 0-15); 618 (69.1%)
176 scored between 0 and 4, 221 (24.7%) between 5 and 9, and 56 (6.3%) between 10 and 15. Of the 52
177 participants with a score of 10 or more at baseline, 31 (59.6%) were no longer severely depressed at
178 six months, of which 30 were still mildly depressed but one dropped to within the ‘normal’ range.
179 The correlation between the GDS-15 score at baseline and at six months was 0.76 (95% confidence
180 interval (CI), 0.73 to 0.79)

## 181 Manipulation of the continuous assignment variable

182 Visual inspection of a histogram of the baseline GDS-15 scores for the 895 participants with available
183 data showed a positively skewed distribution and no clear evidence of manipulation around the cut-
184 point of 10 (**Fig 1**).
185 185
186 ***Fig 1****: Histogram of GDS-15 scores during baseline data collection.*
187 187

## 188 Baseline Characteristics and Testing Continuity

189 Baseline characteristics of the participants by GDS-15 score, are presented in **Table 1**. There are
190 some notable differences between the two groups. For example, the percentages of those with
191 arthritis and those on four or more medications are higher in the group that scored 10 or more on
192 the GDS-15 than those who scored less than 10.
193 ***Table 1****: Participant baseline characteristics stratified by GDS-15 score*

**Baseline GDS-15 Score**

**Characteristic**

**< 10**

**(Not Severely Depressed)**

| **N = 843** | **Depressed)**  **N = 52** | **N = 895** |
| --- | --- | --- |
| **Age (years), mean** 77.7 (7.0)  **(SD)** | 76.0 (7.3) | 77.6 (7.1) |
| **Gender, n (%)** Male ^328^ (38.9) | 27 (51.9) | 542 (60.6) |
| Female 515 (61.1) | 25 (48.1) | 353 (39.4) |
| **Ethnicity, n (%)** White ^836^ (99.2) | 51 (98.1) | 887 (99.1) |
| Other 3 (0.3) | 1 (1.9) | 4 (0.4) |
| **REFORM Treatment** Usual Care ^435^ (51.6) | 26 (50.0) | 461 (51.5) |
| **Group, n (%)** Intervention ^408^ (48.4) | 26 (50.0) | 434 (48.5) |
| **Comorbidity**^✝^**, n (%)** Arthritis ^477^ (56.6) | 40 (76.9) | 517 (57.8) |
| Depression ^61^ (7.2) | 21 (40.4) | 82 (9.2%) |
| Diabetes 268 (31.8) | 26 (50.0) | 294 (32.8) |
| Dizziness/Vertigo ^147^ (17.4) | 22 (42.3) | 169 (18.9) |
| Meniere’s Disease ^25^ (3.0) | 5 (9.6) | 30 (3.4) |
| Numbness in feet ^132^ (15.7) | 15 (28.8) | 147 (16.4) |
| Osteoporosis ^124^ (14.7) | 7 (13.5) | 131 (14.6) |
| **BMI, mean (SD)** 27.6 (5.1) | 30.4 (8.0) | 27.8 (5.4) |
| **≥4 Medications, n (%)** ^496^ (58.8) | 45 (86.5) | 541 (60.4) |
| **Short Falls Efficacy** 11.8 (4.1)  **Scale, mean (SD)** | 16.8 (4.8) | 12.1 (4.3) |
| **I tend to bounce back** Not true at all ^9^ (1.1) | 2 (3.8) | 11 (1.2) |
| **after illness or** Rarely true ^11^ (1.3) | 8 (15.4) | 19 (2.1) |
| **hardship, n(%)** Sometimes true ^115^ (3.6) | 19 (36.5) | 134 (15.0) |
| Often true 248 (29.4) | 17 (32.7) | 265 (29.6) |
| True nearly all of 456 (54.1) the time | 5 (9.6) | 461 (51.5) |
| **I am able to adapt to** Not true at all ^10^ (1.2) | 2 (3.8) | 12 (1.3) |

**≥ 10 (Severely**

**Total**

**change, n (%)**	Rarely true	^20^ (2.4)	5 (9.6)	25 (2.8)

| Sometimes true ^162^ (19.2) | 22 (42.3) | 184 (20.6) |
| --- | --- | --- |
| Often true 255 (30.2) | 20 (38.5) | 275 (30.7) |
| True nearly all of 393 (46.6) the time | 2 (3.8) | 395 (44.1) |
| **Frenchay Activities** 46.9 (7.0)  **Index, mean (SD)** | 39.2 (8.0) | 46.5 (7.2) |
| **EQ-5D-5L score,**  **mean (SD)** 0.7 (0.2) | 0.4 (0.3) | 0.7 (0.2) |
| **Post-16 Education, n** _485_ (57.5)  **(%)** | 26 (50.0) | 511 (57.1) |
| **Degree or Equivalent,** _318_ (37.7)  **n (%)** | 15 (28.8) | 333 (37.2) |
| **Referred to a falls** _52_ (6.2)  **clinic, n (%)** | 4 (7.7) | 56 (6.3) |
| **Have you fallen in the** _402_ (47.7)  **last 6 months?, n (%)** | 30 (57.7) | 432 (48.3) |
| **During the past 4** All of the time ^22^ (2.6) | 12 (23.1) | 34 (3.8) |
| **weeks have you** Most of the time ^51^ (6.0) | 6 (11.5) | 57 (6.4) |
| **worried about having** Good bit of the _71_ (8.4)  **a fall?, n (%)** time | 6 (11.5) | 77 (8.6) |
| Some of the time ^206^ (24.4) | 11 (21.2) | 217 (24.2) |
| Little of the time ^295^ (35.0) | 14 (26.9) | 309 (34.5) |
| None of the time ^193^ (22.9) | 3 (5.8) | 196 (21.9) |
| **Have you broken**  **bones in the past 12** ^55^ (6.5)  **months?, n (%)** | 0 (0.0) | 55 (6.1) |
| **Are you currently**  **wearing an insole or** _295_ (35.0)  **orthotic in your**  **shoe?, n (%)** | 21 (40.4) | 316 (35.3) |
| **Do you require**  **modifications in your** ^113^ (13.4)  **shoes?, n (%)** | 8 (15.4) | 121 (13.5) |
| **Living** Live alone ^361^ (42.8) | 26 (50.0) | 387 (43.2) |
| **Arrangements**^✝^**, n (%)** Live with a partner ^433^ (51.4) | 24 (46.2) | 457 (51.1) |
| Live with a 44 (5.2) friend/relative | 1 (1.9) | 45 (5.0) |
| Sheltered _25_ (3.0) | 3 (5.8) | 27 (3.1) |

accommodation

194 ^✝^ Not mutually exclusive, participants asked to cross all that apply.
195 Continuity was evaluated by comparing participants who scored slightly below the threshold with
196 those who scored slightly above it (**Table 2**). The majority of characteristics were balanced and
197 continuous around the continuous assignment variable cut point. There are a few exceptions such as
198 the difference between groups in the number of participants taking four or more prescribed
199 medications at baseline and EQ-5D-5L score. However, the validity of the regression discontinuity
200 design still holds as we might expect one or two significant results just by chance given the number
201 of comparisons tested.
202 202
203 ***Table 2****: Comparison of characteristics to test balance and continuity between participants who*
204 *scored slightly above and below the continuous assignment variable threshold.*

|  | **Baseline GDS-15 Score** |  |
| --- | --- | --- |
| **8 - 9**  **Characteristic**  **N = 44** | **10 – 11**  **N =27** | **P-value** |
| **Age (years), mean** 78.2 (7.7)  **(SD)** | 77.4 (7.8) | 0.66 |
| **Gender**^✝^**, n (%)** Male ^21^ (47.7)  Female 23 (52.3) | 11 (40.7)  16 (59.3) | 0.57 |
| **Ethnicity**^✝^**, n (%)** White ^44^ (100.0) | 27 (100.0) | N/A |
| **REFORM Treatment** Usual Care ^18^ (40.9)  **Group**^✝^**, n (%)** Intervention ^26^ (59.1) | 12 (44.4)  15 (55.6) | 0.77 |
| **Comorbidity**^✝^**, n (%)** Arthritis ^26^ (59.1) | 20 (74.1) | 0.20 |
| Depression ^10^ (22.7) | 11 (40.7) | 0.11 |
| Diabetes 14 (31.8) | 12 (44.4) | 0.28 |
| Dizziness/Vertigo ^16^ (36.4) | 12 (44.4) | 0.50 |
| Meniere’s Disease 2 (4.6) | 2 (7.4) | 0.61 |
| Numbness in feet ^8^ (18.2) | 9 (33.3) | 0.15 |
| Osteoporosis ^9^ (20.5) | 6 (22.2) | 0.86 |
| **BMI, mean (SD)** 29.9 (5.9) | 29.7 (8.7) | 0.91 |
| **≥4 Medications**^✝^**, n** _30_ (68.2)  **(%)** | 24 (88.9) | 0.05 |
| **Short Falls Efficacy** 14.2 (4.7) | 15.2 (4.4) | 0.35 |

**Scale^a^, mean (SD)**

**I tend to bounce back**	Not true at all	0.0 (0.0)	0.0 (0.0)

**after illness or**	Rarely true	^3^ (6.8)	5 (18.5)

**hardship**^✝^**, n(%)**	Sometimes true	^15^ (34.1)	9 (33.3)

Often true	17 (38.6)	10 (37.0)

0.33

True nearly all of the time

8 (18.2)	2 (7.4)

**I am able to adapt to**	Not true at all	^2^ (4.6)	0 (0.0)

| **change**^✝^**, n (%)** Rarely true ^3^ (6.8)  Sometimes true ^21^ (47.7)  Often true ^11^ (25.0) | 4 (14.8)  9 (33.3)  12 (44.4) | 0.12 |
| --- | --- | --- |
| True nearly all of 7 (15.9) the time | 1 (3.7) |  |
| **Frenchay Activities** 41.6 (7.4)  **Index^b^, mean (SD)** | 41.9 (7.4) | 0.85 |
| **EQ-5D-5L score,**  **mean (SD)** 0.6 (0.2) | 0.4 (0.3) | 0.03 |
| **Post-16 Education**^✝^**, n** _22_ (50.0)  **(%)** | 13 (48.1) | 0.87 |
| **Degree or** _14_ (31.8) | 10 (37.0) | 0.57 |
| **Referred to a falls** _2_ (4.6) | 0 (0.0) | 0.27 |
| **Have you fallen in the**  **last 6 months?** ^✝^**, n** ^23^ (52.3)  **(%)** | 14 (51.9) | 0.97 |
| **During the past 4** All of the time 1 (2.3) | 4 (14.8) |  |
| **weeks have you** Most of the time ^3^ (6.8) | 4 (14.8) |  |
| **worried about having** Good bit of the _12_ (27.3)  **a fall?** ^✝^**, n (%)** time | 2 (7.4) | 0.09 |
| Some of the time ^13^ (29.6) | 5 (18.5) |  |
| Little of the time ^12^ (27.3) | 10 (37.0) |  |
| None of the time ^3^ (6.8) | 2 (7.4) |  |
| **Have you broken**  **bones in the past 12** 3 (6.8) | 0 (0.0) | 0.17 |
| **months?** ^✝^**, n (%) Are you currently** |  |  |
| **wearing an insole or** _21_ (47.7)  **orthotic in your**  **shoe?** ^✝^**, n (%)** | 9 (33.3) | 0.23 |
| **Do you require** 9 (20.5) | 2 (7.4) | 0.14 |

**Equivalent**^✝^**, n (%) clinic**^✝^**, n (%)**

**modifications in your shoes?** ^✝^**, n (%)**

| **Living** Live alone ^20^ (45.5) | 16 (59.3) | 0.26 |
| --- | --- | --- |
| **Arrangements**^✝^**, n (%)** Live with a partner ^21^ (47.7) | 10 (37.0) | 0.38 |
| Live with a 3 (6.8) friend/relative | 0 (0.0) | 0.17 |
| Sheltered _2_ (4.6) | 1 (3.7) | 0.86 |

accommodation

205 ^a^ The higher the score the greater the concern about the possibility of falling
206 ^b^ The higher the score the more often participants carry out some activities i.e. washing up,
207 housework
208 ^✝^ Chi-squared test used; t-test used for all other comparisons.
209 209

## 210 Regression Discontinuity Analysis Results

211 Several functional forms of the regression model were estimated, starting with the simplest linear
212 regression model, which adjusted only for the treatment (GP contacted) and GDS-15 score at
213 baseline and increasing in complexity.
214 The linear regression model with no interaction term, and cubic regression model with an
215 interaction term, produced the lowest AIC value (with and without covariates) (**Table 3**).
216 ***Table 3****: Different functional forms which were estimated, alongside the treatment estimate and AIC*
217 *values.*

| **Functional form** | **Treatment**  **estimate** | **SE, 95% CI** | **p-value** | **AIC** |
| --- | --- | --- | --- | --- |
| *No covariates* |  |  |  |  |
| Linear | -1.08 | 0.38 (-1.83, -0.33) | 0.005 | 3785.7 |
| Linear interaction | -0.84 | 0.46 (-1.74, 0.08) | 0.072 | 3786.8 |
| Quadratic | -0.67 | 0.59 (-1.83, 0.48) | 0.255 | 3786.8 |
| Quadratic interaction | -0.52 | 0.63 (-1.76, 0.72) | 0.411 | 3790.1 |
| Cubic | -0.58 | 0.60 (-1.75, 0.60) | 0.334 | 3788.1 |
| Cubic interaction | 1.08 | 0.91 (-0.71, 2.87) | 0.237 | 3785.5 |
|  |  |  |  |  |
| *With covariates* |  |  |  |  |
| Linear | -1.02 | 0.38 (-1.77, -0.27) | 0.008 | 3772.2 |
| Linear interaction | -0.83 | 0.47 (-1.75, 0.08) | 0.074 | 3773.7 |
| Quadratic | -0.71 | 0.59 (-1.87, 0.45) | 0.230 | 3773.7 |

| Quadratic interaction | -0.54 | 0.64 (-1.80, 0.70) | 0.389 | 3777.0 |
| --- | --- | --- | --- | --- |
| Cubic | -0.63 | 0.60 (-1.81, 0.55) | 0.294 | 3775.2 |
| Cubic interaction | 0.99 | 0.92 (-0.80, 2.79) | 0.277 | 3772.8 |

218 218
219 Again, starting with the simplest linear regression model, an F-test was calculated and was not found
220 to be statistically significant (p=0.40). This suggests that the simple linear regression model
221 adequately depicts the relationship between the outcome and rating variables and therefore can
222 serve as an appropriate choice for the RDD estimation model as opposed to a more complex model.
223 **Fig 2** shows a scatter plot of participants’ GDS-15 score at six months against their GDS-15 score at
224 baseline with the fitted regression line from the selected model. There is a noticeable drop in the
225 regression line at the cut-point, with those scoring 10 or higher on the GDS-15 at baseline scoring
226 less on the GDS-15 at six months than expected, with a clear discontinuity present. The regression
227 coefficient of interest is -1.08 (95% CI -1.83 to -0.33, p=0.005), which indicates that participants who
228 scored 10 or more and whose GP was subsequently contacted had a just over one-point reduction in
229 depression severity six months later.
230 ***Fig 2****: Scatter plots of participant’s GDS-15 score at six-months against their GDS-15 score at*
231 *baseline, with the fitted values of the selected linear regression model.*
232 The robustness of the linear regression model and sensitivity of the treatment estimate was
233 assessed by dropping the outermost 1% of the data and fitting the linear regression model again.
234 When discarding the outermost 1%, the treatment estimate becomes -1.23 (SE 0.43, 95% CI -2.09 to
235 -0.38), an absolute change of 0.15.

## 236 Discussion

237 Depression among older people is a large source of morbidity in the community. There has been no
238 large RCT to test the effectiveness of primary care case finding among the general population of
239 older people in the UK. In this study we undertook a regression discontinuity analysis of people aged
240 65 years and above who were registered with local NHS podiatry services and had at least one risk
241 factor for falling. As part of the REFORM trial we gave participants the GDS-15 to complete at
242 baseline and six months. At baseline, 29% of participants had GDS-15 scores indicating they were at
243 least mildly depressed, with 6% exhibiting symptoms of severe depression (score of 10 or more).
244 These figures are in line with national findings that around a quarter of older people suffer from
245 depression [2]. For ethical reasons we contacted the GPs of all participants who scored 10 points or
246 above on the GDS-15 at enrolment to the REFORM trial. Following completion of the study, we
247 realised that these data were appropriate to conduct a retrospective regression discontinuity
248 analysis, and observed that, after six months, this intervention resulted in a significant improvement
249 in the GDS-15 score, suggesting that a form of routine screening using the GDS-15 might be effective
250 at reducing the severity of depression in older people.
251 These findings differ from those of a recent cluster RCT in the US to determine the effect of case-
252 finding for depression, which was unable to demonstrate any benefit [30]. Thirteen primary care
253 medical clinics were randomised to intervention and control. As in our study, the GDS-15 was used
254 to measure depressive symptoms and, for patients in the intervention group, their physician was
255 informed if they scored over a certain threshold. The threshold was set at six, which was lower than
256 our threshold, and different interventions were recommended to be offered to people scoring 6-10
257 and those scoring 11-15. There was no statistically significant difference in mean GDS-15 score at the
258 two year follow-up.
259 259

# 260 Strengths and Limitations

261 There are a number of limitations to this study. First, all of our participants had podiatry needs and
262 were at high risk of falling and so we cannot be sure our results will generalise to all people aged
263 over 65 living in the community. Another limitation is that we are unsure exactly what intervention
264 GPs gave to patients that led to an improvement in care. We assumed that GPs followed best
265 professional practice, which may have included a consultation with the participant to discuss their
266 symptoms and subsequent management of their depression with medication and/or psychological
267 therapy, but in any future study it would be important to get a detailed description of what
268 treatments were offered to (and accepted by) patients.
269 Further there are limitations to the analysis design. This was an opportunistic analysis embedded
270 within an RCT; therefore the sample size was constrained and not calculated specifically for this
271 analysis. Generally, to answer the same research question, an RDD requires a much larger sample
272 size than an RCT, at least three to five times more participants [31, 32]. There was an approximately
273 1.1 point GDS-15 score reduction at the cut-point and whether this is clinically meaningful is
274 uncertain; however, previous studies have utilised a similar (1.2 point) change in the GDS-15 as a
275 clinically important difference [33]. The parametric approach to a regression discontinuity design
276 requires the selection of a model that best fits the data. Different techniques to select the final
277 model may give different results. Here, when we considered the lowest AIC, there was little
278 difference between the linear regression and cubic regression with an interaction; however these
279 gave treatment effects that were qualitatively different, similar in magnitude but in the opposite
280 direction. When the final model is specified, one sensitivity check that is suggested includes
281 repeating the analysis with the outermost 1%, 5% and 10% data points excluded. However given the
282 small sample size here and that only 6% of participants scored above the cut-point, we were
283 restricted on the robustness checks that could be carried out. The treatment estimate when the
284 outermost 1% of the data are dropped increased in magnitude, which may cast further doubt that
285 the functional form (the simplest linear regression model) is correctly specified.
286 On the other hand, a strength of the regression discontinuity design is the way in which a treatment
287 effect can be visualised. The discontinuity or drop in the regression line makes it clear if a treatment
288 effect is or is not present.
289 Another strength of the study is the generalisability to a wide number of GP surgeries. The 52
290 participants with a GDS-15 baseline score of 10 or more were recruited from 43 GP surgeries, which
291 suggests that the effect, if true, is not driven by the management strategies of a ‘few’ GPs and is
292 likely to be representative of the impact of typical GP care.
293 In summary, a serendipitous evaluation suggests that routine case finding for depression with the
294 GDS-15 may improve the mental wellbeing of older people. Given the promise indicated from such a
295 simple intervention, the feasibility, acceptability, effectiveness, and cost-effectiveness of a well-
296 designed case-finding approach is worthy of further exploration. In addition, our exemplar
297 demonstrates how a regression discontinuity analysis can be conducted retrospectively or
298 prospectively embedded in other studies to answer a further research question, or at least to
299 generate plausible future research hypotheses.

# 300 Acknowledgments

301 We thank the REFORM trial participants for their participation in the REFORM trial and providing
302 outcome data.

# 303 References

304 1.	Members of executive of Faculty of Old Age Psychiatry, t.R.C.o.P., *Suffering in silence: age*
305 *inequality in older people's mental health care*. 2018.
306 2.	Health, D.o., *No health without mental health: A cross-government mental health outcomes*
307 *strategy for people of all ages.* Supporting document–the economic case for improving
308 efficiency and quality in mental health, 2011.
309 3.	Age, U., *Hidden in plain sight: The unmet mental health needs of older people.* Age UK,
310 London, available at: [www.](http://www/) ageuk. org.
311 uk/brandpartnerglobal/wiganboroughvpp/hidden_in_plain_sight_older_
312 peoples_mental_health. pdf (accessed 19 February 2019), 2016.
313 4.	Chew-Graham, C., R. Baldwin, and A. Burns, *Treating depression in later life*. 2004, British
314 Medical Journal Publishing Group.
315 5.	Ferenchick, E.K., P. Ramanuj, and H.A. Pincus, *Depression in primary care: part 1—screening*
316 *and diagnosis.* BMJ, 2019. **365**.
317 6.	Fiske, A., J.L. Wetherell, and M. Gatz, *Depression in older adults.* Annual review of clinical

318	psychology, 2009. **5**: p. 363-389.

319 7.	Siu, A.L., et al., *Screening for depression in adults: US Preventive Services Task Force*
320 *recommendation statement.* Jama, 2016. **315**(4): p. 380-387.
321 8.	Excellence, N.I.f.C., *Depression in adults: Recognition and management. Clinical guideline*
322 *[CG90]*. 2009, NICE London.
323 9.	Mallen, C., et al., *The effects of implementing a point-of-care electronic template to prompt*
324 *routine anxiety and depression case-finding in patients consulting for clinical osteoarthritis*
325 *(the POST trial): a cluster randomised trial in primary care.* PLoS Med, 2017.
326 10.	Kendrick, T. and E. Maund, *Do PROMS improve outcomes in patients with depression in*
327 *primary care?* bmj, 2020. **370**.
328 11.	Cockayne, S., et al., *Cohort randomised controlled trial of a multifaceted podiatry*
329 *intervention for the prevention of falls in older people (the REFORM trial).* PLoS One, 2017.

330	**12**(1): p. e0168712.

331 12.	Sheikh, J.I. and J.A. Yesavage, *Geriatric Depression Scale (GDS): recent evidence and*
332 *development of a shorter version.* Clinical Gerontologist: The Journal of Aging and Mental
333 Health, 1986.
334 13.	Brink, T.L., et al., *Screening tests for geriatric depression.* Clinical gerontologist, 1982. **1**(1): p.

335	37-43.

336 14.	Yesavage, J.A., et al., *Development and validation of a geriatric depression screening scale: a*
337 *preliminary report.* Journal of psychiatric research, 1982. **17**(1): p. 37-49.
338 15.	Wancata, J., et al., *The criterion validity of the Geriatric Depression Scale: a systematic*
339 *review.* Acta Psychiatrica Scandinavica, 2006. **114**(6): p. 398-410.
340 16.	Boey, K.W., *The use of GDS-15 among the older adults in Beijing.* Clinical Gerontologist,

341	2000. **21**(2): p. 49-60.

342 17.	Brown, P.J., C.M. Woods, and M. Storandt, *Model stability of the 15-item Geriatric*
343 *Depression Scale across cognitive impairment and severe depression.* Psychology and aging,

344	2007. **22**(2): p. 372.

345 18.	Lesher, E.L. and J.S. Berryhill, *Validation of the geriatric depression scale‐short form among*
346 *inpatients.* Journal of clinical psychology, 1994. **50**(2): p. 256-260.
347 19.	Smalbrugge, M., et al., *Screening for depression and assessing change in severity of*
348 *depression. Is the Geriatric Depression Scale (30-, 15-and 8-item versions) useful for both*
349 *purposes in nursing home patients?* Aging and Mental Health, 2008. **12**(2): p. 244-248.
350 20.	Olin, J.T., et al., *The Geriatric Depression Scale and the Beck Depression Inventory as*
351 *screening instruments in an older adult outpatient population.* Psychological Assessment,

352	1992. **4**(2): p. 190.

353 21.	Low, G.D. and A.M. Hubley, *Screening for depression after cardiac events using the Beck*
354 *Depression Inventory-II and the Geriatric Depression Scale.* Social Indicators Research, 2007.

355	**82**(3): p. 527.

356 22.	Lyness, J.M., et al., *Screening for depression in elderly primary care patients: A comparison of*
357 *the Center for Epidemiologic Studies—Depression Scale and the Geriatric Depression Scale.*
358 Archives of internal medicine, 1997. **157**(4): p. 449-454.
359 23.	Scogin, F., *The concurrent validity of the Geriatric Depression Scale with depressed older*
360 *adults.* Clinical Gerontologist, 1987. **7**(1): p. 23-32.
361 24.	Dunn, V.K. and W.P. Sacco, *Psychometric evaluation of the Geriatric Depression Scale and the*
362 *Zung Self-Rating Depression Scale using an elderly community sample.* Psychology and Aging,

363	1989. **4**(1): p. 125.

364 25.	Chen, S., et al., *Impact of community based screening for hypertension on blood pressure*
365 *after two years: regression discontinuity analysis in a national cohort of older adults in China.*

366	bmj, 2019. **366**: p. l4064.

367 26.	O’Keeffe, A.G., et al., *Regression discontinuity designs: an approach to the evaluation of*
368 *treatment efficacy in primary care using observational data.* Bmj, 2014. **349**.
369 27.	Venkataramani, A.S., J. Bor, and A.B. Jena, *Regression discontinuity designs in healthcare*

370	*research.* bmj, 2016. **352**: p. i1216.

371	28.	Lee, D.S. and T. Lemieux, *Regression discontinuity designs in economics.* Journal of economic

372	literature, 2010. **48**(2): p. 281-355.

373 29.	Sood, N., et al., *Government health insurance for people below poverty line in India: quasi-*
374 *experimental evaluation of insurance and health outcomes.* Bmj, 2014. **349**: p. g5114.
375 30.	Whooley, M.A., B. Stone, and K. Soghikian, *Randomized trial of case‐finding for depression in*
376 *elderly primary care patients.* Journal of General Internal Medicine, 2000. **15**(5): p. 293-300.
377 31.	Deke, J. and L. Dragoset, *Statistical Power for Regression Discontinuity Designs in Education:*
378 *Empirical Estimates of Design Effects Relative to Randomized Controlled Trials. Working*
379 *Paper.* Mathematica Policy Research, Inc., 2012.
380 32.	Schochet, P.Z., *Statistical power for regression discontinuity designs in education*
381 *evaluations.* Journal of Educational and Behavioral Statistics, 2009. **34**(2): p. 238-266.
382 33.	Vinkers, D.J., et al., *The 15‐item Geriatric Depression Scale (GDS‐15) detects changes in*
383 *depressive symptoms after a major negative life event. The Leiden 85‐plus Study.*
384 International journal of geriatric psychiatry, 2004. **19**(1): p. 80-84.
385 385

Figure 1	[Click here to access/download;Figure;Fig1.tif](https://www.editorialmanager.com/pone/download.aspx?id=32654586&guid=3e20c4e5-b3c7-4b0c-9ef2-ddc05bf89e80&scheme=1)

Figure 2	[Click here to access/download;Figure;Fig2.tif](https://www.editorialmanager.com/pone/download.aspx?id=32654587&guid=744f6046-7e23-43d9-88ab-8fdfc7c3e3a0&scheme=1)

S1 Appendix A

[Click here to access/download](https://www.editorialmanager.com/pone/download.aspx?id=32654589&guid=fe81b9b1-b2b7-4431-8c54-d4a7022d5789&scheme=1)

[**Supporting Information**](https://www.editorialmanager.com/pone/download.aspx?id=32654589&guid=fe81b9b1-b2b7-4431-8c54-d4a7022d5789&scheme=1)

[S1 Appendix A. Geriatric Depression Scale (15- item)..docx](https://www.editorialmanager.com/pone/download.aspx?id=32654589&guid=fe81b9b1-b2b7-4431-8c54-d4a7022d5789&scheme=1)
